# Physical activity to improve cognition in older adults: can physical activity programs enriched with cognitive challenges enhance the effects? A systematic review and meta-analysis

**DOI:** 10.1186/s12966-018-0697-x

**Published:** 2018-07-04

**Authors:** Freja Gheysen, Louise Poppe, Ann DeSmet, Stephan Swinnen, Greet Cardon, Ilse De Bourdeaudhuij, Sebastien Chastin, Wim Fias

**Affiliations:** 1 Department of Movement and Sports Sciences, Ghent University, Ghent, Belgium; 2 Movement Control and Neuroplasticity Research Group, KU Leuven, Leuven, Belgium; 3 Institute of Applied Health Research, School of Health and Life Science, Glasgow Caledonian University, Glasgow, UK; 4 Department of Experimental Psychology, Ghent University, Ghent, Belgium

**Keywords:** Meta-analysis, Older adults, Physical activity, Cognitive activity, Cognition, Combined intervention, Cognitive impairment

## Abstract

**Background:**

Aging-related cognitive decline and cognitive impairment greatly impacts older adults’ daily life. The worldwide ageing of the population and associated wave of dementia urgently calls for prevention strategies to reduce the risk of cognitive decline. Physical activity (PA) is known to improve cognitive function at older age through processes of neuroplasticity. Yet, emerging studies suggest that larger cognitive gains may be induced when PA interventions are combined with cognitive activity (CA). This meta-analysis evaluates these potential synergistic effects by comparing cognitive effects following combined PA + CA interventions to PA interventions (PA only), CA interventions (CA only) and control groups.

**Methods:**

Pubmed, Embase, PsycInfo, CINAHL and Sportdiscus were searched for English peer-reviewed papers until April 2018. Data were extracted on cognition and factors potentially influencing the cognitive effects: mode of PA + CA combination (sequential or simultaneous), session frequency and duration, intervention length and study quality. Differences between older adults with and without mild cognitive impairments were also explored.

**Results:**

Forty-one studies were included. Relative to the control group, combined PA + CA intervention showed significantly larger gains in cognition (*g* = 0.316; 95% CI 0.188–0.443; *p* < .001). Studies that compared combined PA + CA with PA only, showed small but significantly greater cognitive improvement in favor of combined interventions (*g* = 0.160; 95% CI 0.041–0.279; *p* = .008). No significant difference was found between combined PA + CA and CA only interventions. Furthermore, cognitive effects tended to be more pronounced for studies using simultaneous designs (*g* = 0.385; 95%CI 0.214–0.555; *p* < .001) versus sequential designs (*g* = 0.114; 95%CI -0.102- 0.331, *p* = .301). Effects were not moderated by session frequency, session duration, intervention length or study quality. Also, no differences in effects were found between older adults with and without mild cognitive impairments.

**Conclusion:**

Findings of the current meta-analysis suggest that PA programs for older adults could integrate challenging cognitive exercises to improve cognitive health. Combined PA + CA programs should be promoted as a modality for preventing as well as treating cognitive decline in older adults. Sufficient cognitive challenge seems more important to obtain cognitive effects than high doses of intervention sessions.

**Electronic supplementary material:**

The online version of this article (10.1186/s12966-018-0697-x) contains supplementary material, which is available to authorized users.

## Background

Cognitive impairment including dementia is now the leading cause of disablement and death in later life. Worldwide, 47 million people are living with dementia and this will almost triple by 2050 [1]. The decline in cognitive function impacts individuals as well as their families and significantly reduces independence, quality of life and daily life functional abilities. Today we are in front of unprecedented pressure from ageing of the population and the associated tidal wave of dementia to develop prevention strategies that reduce the risk of cognitive decline.

Recent literature suggests that cognitive decline is preventable as the brain retains plasticity in later life [2, 3]. Physical activity (PA, especially aerobic and strength exercise) is known to play an important role in the protection against cognitive decline and dementia [4–6] through processes of neuroplasticity. PA intervention studies in older adults have demonstrated effects on brain structure, function and connectivity [7–10]. Yet, novel evidence suggests that enriching PA interventions with cognitive challenge might maximize the neuroplastic properties of the brain that could enhance the potential of prevention and treatment programs for alleviating cognitive decline.

Laboratory animal studies have shown that combining PA with cognitive training induces larger effects on neurocognitive functioning than PA interventions alone [11–13]. Two systematic reviews [14, 15] and two meta-analyses [16, 17] suggest that this might also be the case in humans and that combined physical and cognitive training (PA + CA) strategies could be used as a modality to improve cognition in older adults. Yet, many research questions remain. To date, the superiority of combined PA + CA interventions over PA interventions alone and CA interventions alone is still questionable and calls for more evidence from well-performed interventions studies. Also, there is currently a huge gap in knowledge with respect to how such interventions should be delivered and whether this modality can also be used for treating cognitive decline in a population with already some mild cognitive impairment.

So far, the evidence for combined PA + CA reported in previous reviews [14–17] rests on controlled, rather artificially designed dual-tasking exercises (e.g. walking and learning new sequences of word lists) difficult to introduce into sustainable real world interventions. The question remains whether existing activity programs such as dance and tai chi that have a stronger inherent nature of combined physical and cognitive training can also elicit cognitive benefits. Dancing has been reported to be an enjoyable activity leading to high adherence rates and typically requires a simultaneous engagement of both endurance and coordination as well as executive functions, learning and memorizing new step sequences [18, 19]. Likewise, tai chi or related martial arts offer a unique combination of moderate-intense aerobic exercise [20] with cognitive training: learning new sequences of movement patterns which involve e.g., visuospatial processing, episodic memory and attentional control [21, 22]. The current meta-analysis integrates evidence from more experimental dual-tasks with that on interventions with more naturally combined PA + CA (such as dance and tai chi programs) to better understand if combined PA + CA training is more beneficial than PA or CA interventions alone.

Furthermore, the current meta-analysis explores potential dose-response relationships. An important question is whether the cognitive effects of combined PA + CA interventions are influenced by the frequency with which the sessions are delivered or might be influenced by the duration of the sessions and/or the total length of the intervention. Another crucial question is whether PA and CA should be delivered simultaneously or as separate sessions one after the other (i.e. sequentially). To induce interactive, synergistic cognitive effects, it has been suggested that both activities are preferably conducted simultaneously [11]. This is because the effect of PA on neuroplasticity facilitation (e.g. the release of neurotrophic factors) is restricted in time and returns to baseline 10–60 min after the physical activity [23]. Yet the evidence from combined PA + CA human intervention studies so far was inconclusive with respect to this latter question [16].

Finally it is important to understand if combined PA + CA programs can be used only as a preventive modality or whether it also has beneficial treatment effect such as improving cognition in cognitively impaired older adult population. A recent meta-analysis on exergame studies reported cognitive benefits for clinical populations with conditions related to neurocognitive impairments [17]. The current meta-analysis further explores potential differences in cognitive effects between healthy older adults and older adults with mild cognitive impairments, now also including more naturally combined PA + CA such as dance and tai chi programs.

## Methods

This work complies with the Preferred Reporting Items for Systematic Reviews and Meta-analysis (PRISMA) guidelines (see Additional file 1). The study protocol is detailed in Additional file 2.

### Eligibility criteria

Studies were considered eligible when fulfilling the following criteria:

- *Study population:* Independently living older adults (overall mean age ≥ 65 years) with or without mild cognitive impairments at baseline but without dementia, and without other mental health issues or neurological disease (e.g. stroke, depression, parkinson).
- *Combined PA + CA intervention:* PA sessions including aerobic or strength training components or a combination of both. CA sessions must involve cognitive training exercises aimed to train single or multiple domains of cognitive function. The combination could be *sequential* with separate sessions of PA before or after separate sessions of CA or *simultaneous* with sessions including PA and CA concurrently through for instance exergames, dual-task exercises, dance, tai chi or related martial arts. Studies were only included when they were intentionally designed and clearly described as a multimodal physically and cognitively effortful intervention. For instance, when dance or tai chi classes were described rather as social activities and had no explicit focus on continuous physical stimulation and new cognitive learning/training elements, such studies were excluded.
- *Comparison interventions:* Studies were only considered when at least one of the following comparison groups was included: passive or active control group (e.g. no intervention/usual care or classes comprising e.g. stretching exercises, i.e. not comprising any aerobic/strength training or cognitive training), PA only group (including aerobic (e.g. walking, cycling) or strength training (e.g. leg presses, seated rowing) or a combination of both) or CA only group (e.g. computer based cognitive games, memorization or visual search tasks). When there were some CA training elements in the PA only group or PA training elements in the CA only group these comparison groups were not included in the analyses.
- *Study outcomes*: objectively measured cognitive functions (e.g. memory, attention, executive control)
- *Study design*: pre-post intervention trials with comparison group; randomized controlled trial (RCT), cluster-RCT, non-randomized controlled trial. Studies evaluating the effects of a single bout of exercise were not considered. Studies were also excluded when the combined PA + CA intervention included an additional lifestyle intervention (e.g. diet or dietary supplements, psychological group counseling).

### Search strategy and study selection

Pubmed, Embase, PsycInfo, CINAHL and Sportdiscus were searched for English peer-reviewed publications since the start of the database until the 20th of April 2018.Keywords were used including a combination of PA search terms (e.g., physical OR aerobic OR strength) AND CA terms (cognitive OR mental OR mind) OR terms related to combination of elements (e.g., multimodal OR tai chi OR dual task OR dance OR exergame) together with terms related to intervention designs (e.g., training OR exercise OR program) AND terms related to older adult participants (e.g., aging OR senior OR mild cognitive impairment) AND terms related to cognition (e.g., cognitive OR memory OR executive control). See Additional file 2 for full search terms. References and citation lists of papers and published reviews were additionally searched. Authors were contacted when necessary data for effect size calculation (raw cognitive outcome measures and/or positive/negative scoring of cognitive tests) were missing. Initial screening based on title and abstract was performed by the first author (FG). After this first selection full texts were screened independently by two reviewers (FG and LP) in accordance with the eligibility criteria set forth in the study protocol. Consensus was used to resolve disagreement regarding inclusion of the studies. When doubt regarding study eligibility persisted, this was resolved by a third reviewer (WF).

### Data extraction and analysis

Data extraction (cognitive outcomes, moderator variables) was done by FG and LP independently. Consensus was used to resolve disagreement; when doubt persisted this was resolved by including a third reviewer (WF). For the meta-analyses, cognitive outcome data were extracted in the form of means and standard deviations of each group for both pre and post assessment (or mean changes and SD differences or F values for group differences between changes). An effect size was calculated for each study with Hedges’ formula correcting for small samples [24]. Comprehensive Meta-analysis (CMA) software version 3.3.070 (Biostat Inc., Englewood, NJ, USA) was used to compute effect sizes and conduct all analyses. For each study, effect sizes were averaged across all cognitive measures to determine the effect of combined PA + CA intervention versus the comparison groups on overall cognition. Random effects models were used with a positive Hedges’ g or a negative Hedges’ g indicating that the combined PA + CA intervention induced respectively higher or lower gains in cognition versus the comparison intervention. Where the cognitive outcome measure was negatively scored (higher scores reflect decline of cognitive function), the computed sign of the effect size was reversed so all positive differences reflected a higher improvement in cognition for the combined PA + CA intervention than for the comparison group. Heterogeneity between studies was assessed using the Cochran’s Q-value and I^2^ statistic with a significant *p* value indicating large variability of effect sizes between studies. Moderator analyses were conducted to test whether the heterogeneity could be explained by differences in session duration, session frequency, intervention length, cognitive status of participants (cognitively healthy versus cognitively impaired) and mode of combination (sequential versus simultaneous). Moderator analysis also evaluated whether study quality (3 categories: weak-moderate-strong) explained differences in effect sizes. The quality of each study was evaluated independently by two raters (LP and AD) using the Effective Public Health Practice Project (EPHPP) assessment tool for public health interventions (https://merst.ca/ephpp/) (see Additional file 3). FG was consulted to resolve disagreement and reach a consensus quality score.

### Sensitivity analyses

Additional analyses were performed to identify possible outliers (mean Hedges’ g effect size ±3SD). All analyses (combined PA + CA versus control; combined PA + CA versus PA only; combined PA + CA versus CA only) were repeated for randomized controlled trials only (i.e., excluding non-randomized controlled trials) and for pre–posttest correlations set at lower (0.20) and higher (0.80) values than the standard assumption of 0.50. Finally, potential publication bias was evaluated via a funnel plot and Egger’s regression test.

## Results

### Included studies

**Fig. 1:**
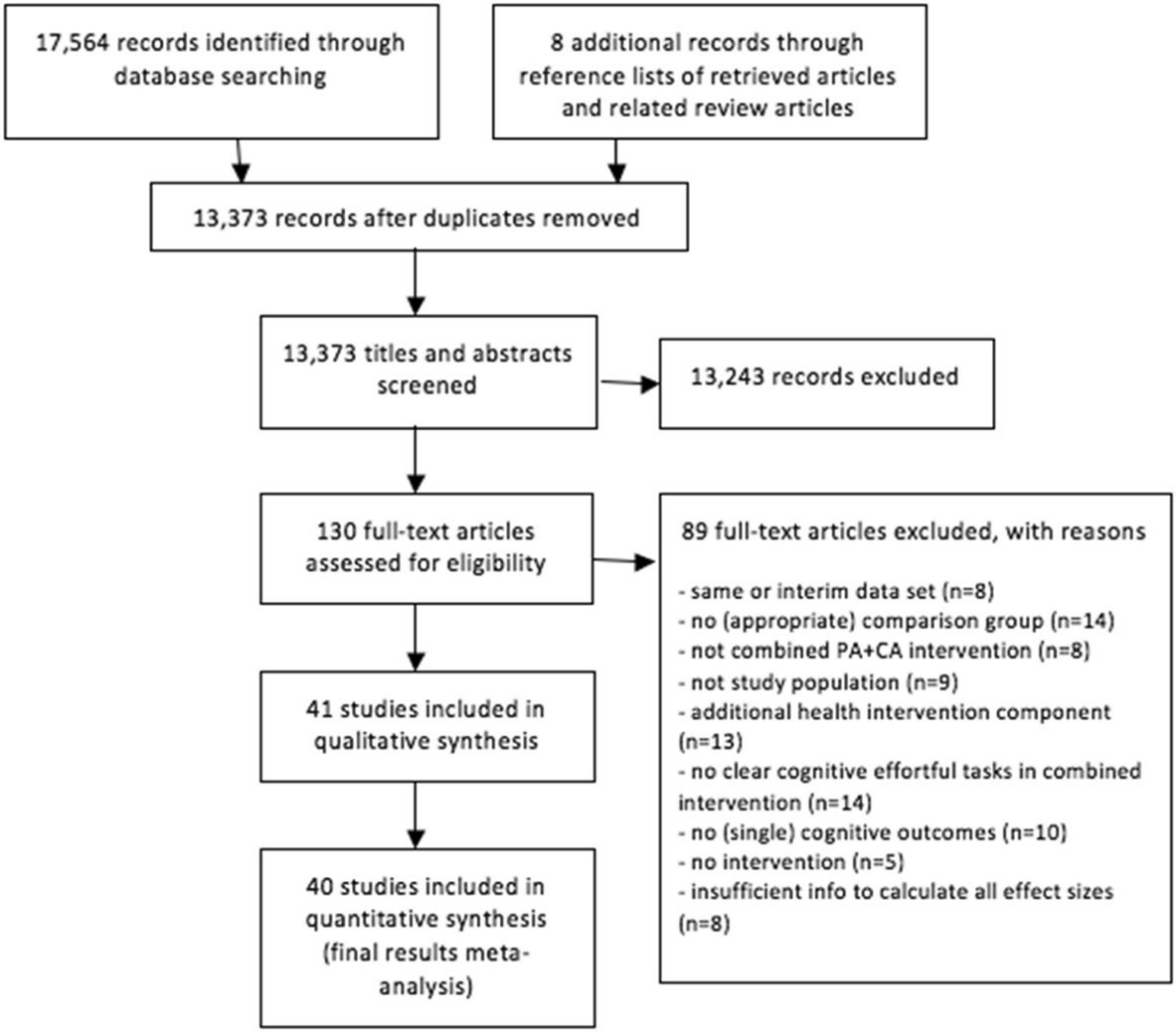
Flow chart of study selection process

The flow of the study selection process is summarized in Fig. 1. Altogether, 41 articles were included in the review (see Table 1 for an overview and characteristics of included studies) [19, 21, 25–63]. Nine studies combined PA and CA sessions using a sequential design; 29 studies used a simultaneous design and three studies had both sequential and simultaneous components. Simultaneously integrated PA + CA interventions consisted of exergames (*n* = 6), dance (*n* = 5), tai chi (*n* = 6), karate (*n* = 2), or dual-tasks (*n* = 12), and one study included both an exergame and dual-task program. Thirty studies included a population with cognitively healthy older adults. Eleven studies included a population with older adults with mild cognitive impairments (MCI). Out of these eleven studies, seven studies [31, 33, 35, 39, 51, 52, 63] included a population with a diagnosis of MCI based on the commonly used criteria of Petersen (2004), i.e., memory impairment based on both subjective and objective testing, in the absence of dementia and without significant loss of daily functioning [64]. Three studies [41, 58, 62] defined cognitive impairment based on Mini-Mental State Examination (MMSE) criteria only, and another study [26] included older adults with cognitive complaints based on self-reports. In this meta-analysis, the latter study was also categorized as a study on a cognitively impaired population. Previous neuroimaging studies have demonstrated that older adults with cognitive complaints display changes in grey matter density and white matter integrity that are similar to older adults with diagnosed MCI; patterns of brain changes that are different from cognitively healthy older adults [65, 66].

**Table 1:** Characteristics of included studies

Authors	Year	Country	Mode of PA + CA combination	Type of sim	Comparison groups	Cognitive status	Intervention length	Session duration	Session frequency	EPHPP study quality	Study design
Fabre et al.	2002	France	seq	n/a	control, PA, CA	healthy	short	long	high	weak	RCT
Oswald et al.	2006	Germany	seq	n/a	control, CA^a^	healthy	long	long	low	weak	non-RCT
Taylor-Piliae et al.	2010	US	sim	tai chi	control, PA	healthy	long	n/a	high	weak	RCT
Kim et al.	2011	South Korea	sim	dance	control	healthy	long	medium	medium	strong	non-RCT
Legault et al.	2011	US	seq	n/a	control, PA, CA	healthy	medium	n/a	n/a	weak	RCT
Hiyamizu et al.	2012	Japan	sim	dual-task	PA	healthy	medium	medium	medium	strong	RCT
Jansen et al.	2012	Germany	sim	karate	control, PA, CA	healthy	medium	medium	n/a	moderate	non-RCT
Maillot et al.	2012	France	sim	exergame	control	healthy	medium	medium	medium	moderate	RCT
Lam et al.	2012	Hong Kong	sim	tai chi	control	impaired	long	short	high	strong	cRCT
Barnes et al.	2013	US	seq	n/a	PA^b^	impaired	medium	medium	high	moderate	RCT
Kattenstroth et al.	2013	Germany	sim	dance	control	healthy	long	medium	low	weak	RCT
Schoene et al.	2013	Australia	sim	exergame	control	healthy	short	short	medium	moderate	RCT
Suzuki et al.	2013	Japan	sim	dual-task	control	impaired	long	long	medium	strong	RCT
Theill et al.	2013	Switzerland	sim	dual-task	control, CA	healthy	short	short	medium	weak	non-RCT
Teixeira et al.	2013	Brazil	sim	dual-task	control	healthy	medium	short	high	moderate	non-RCT
Fiatarone et al.	2014	Australia	seq	n/a	control, PA, CA	impaired	long	long	medium	moderate	RCT
Hughes et al.	2014	US	sim	exergame	control	impaired	long	long	low	strong	RCT
Shah et al.	2014	Australia	seq	n/a	control, PA, CA	healthy	medium	medium	high	moderate	non-RCT
van het Reve et al.	2014	Switzerland+Germany	seq	n/a	PA	healthy	medium	short	high	weak	RCT
Li et al.	2014	US	sim	tai ji quan	control	impaired	medium	medium	medium	moderate	non-RCT
Hackney et al.	2015	US	sim	dance	control	healthy	medium	long	n/a	strong	non-RCT
Eggenberger et al.	2015	Switzerland	sim	exergame and dual task^c^	PA	healthy	long	medium	medium	weak	RCT
Yokoyama et al.	2015	Japan	sim	dual-task	PA	healthy	medium	medium	high	moderate	RCT
Sato et al.	2015	Japan	sim	dual-task	PA	healthy	short	medium	low	moderate	RCT
Nishiguchi et al.	2015	Japan	sim + seq	dual-task	control	healthy	medium	long	n/a	strong	RCT
Styliadis et al.	2015	Greece	sim + seq	exergame	control, CA^a,d^	impaired	short	medium	high	weak	non-RCT
Kitazawa et al.	2015	Japan	sim	dual-task	control	healthy	short	medium	low	moderate	RCT
Leon et al.	2015	Spain	sim	dual-task	control, PA	healthy	medium	medium	medium	moderate	RCT
Ansai et al.	2016	Brazil	sim	dual-task	PA	healthy	medium	medium	high	weak	cRCT
Desjardins-Crépeau et al.	2016	Canada	seq	n/a	control, PA, CA	healthy	medium	medium	high	moderate	RCT
Eggenberger et al.	2016	Switzerland	sim	exergame	control	healthy	short	short	high	weak	RCT
Falbo et al.	2016	Italy	sim	dual-task	PA	healthy	medium	medium	medium	strong	RCT
Hagovska et al.	2016	Slovac Republic	sim + seq	dual-task	PA	impaired	short	short	high	strong	RCT
Lu et al.	2016	Hong Kong	sim	tai chi	control	healthy	medium	long	high	moderate	RCT
Witte et al.	2016	Germany	sim	karate	control, PA	healthy	medium	medium	medium	moderate	RCT
Merom et al.	2016	Australia	sim	dance	PA	healthy	long	medium	medium	moderate	RCT
Schättin et al.	2016	Switzerland	sim	exergame	control	healthy	short	short	high	moderate	RCT
Müller et al.	2017	Germany	sim	dance	PA	healthy	long	long	medium	weak	RCT
Sungkarat et al.	2017	Thailand	sim	tai chi	control	impaired	medium	medium	high	strong	RCT
Damirchi et al.	2018	Iran	seq	n/a	control, PA, CA	impaired	short	medium	high	moderate	RCT
Siu et al.	2018	Hong Kong	sim	tai chi	control	impaired	medium	medium	medium	weak	cRCT

*seq* Sequential, *sim* Simultaneous, *PA* Only physical activity program, *CA* Only cognitive activity program, intervention length: short (< 12 weeks), medium (12–23 weeks), or long (≥ 24 weeks); session duration: short (≤ 45 min), medium (> 45 to ≤60 min) or long (> 60 min); session frequency: low (1 session/week), medium (2 sessions/week) or high (≥ 3 sessions/week). *RCT* Randomized controlled trial, *non-RCT* Non-randomized controlled trial, *cRCT* Cluster randomized controlled trial

^a^the comparison with the PA group was removed from present meta-analysis because this program also included forms of cognitive training which was a priori defined as a potential bias in our protocol

^b^the CA and control group were removed from present meta-analysis because these programs also included strength training which was a priori defined as a potential bias in our protocol

^c^effect sizes were combined for both simultaneous programs following Higgings and Green, 2011 (cf. study protocol)

^d^the active (not passive) control group was included (cf. study protocol)

n/a = type of simultaneous combination could not be defined given the sequential design, or moderator variable could not be clearly defined and hence, was removed from moderator analysis

### Overall effects on cognitive function

#### Combined PA + CA versus control group

**Fig. 2:**
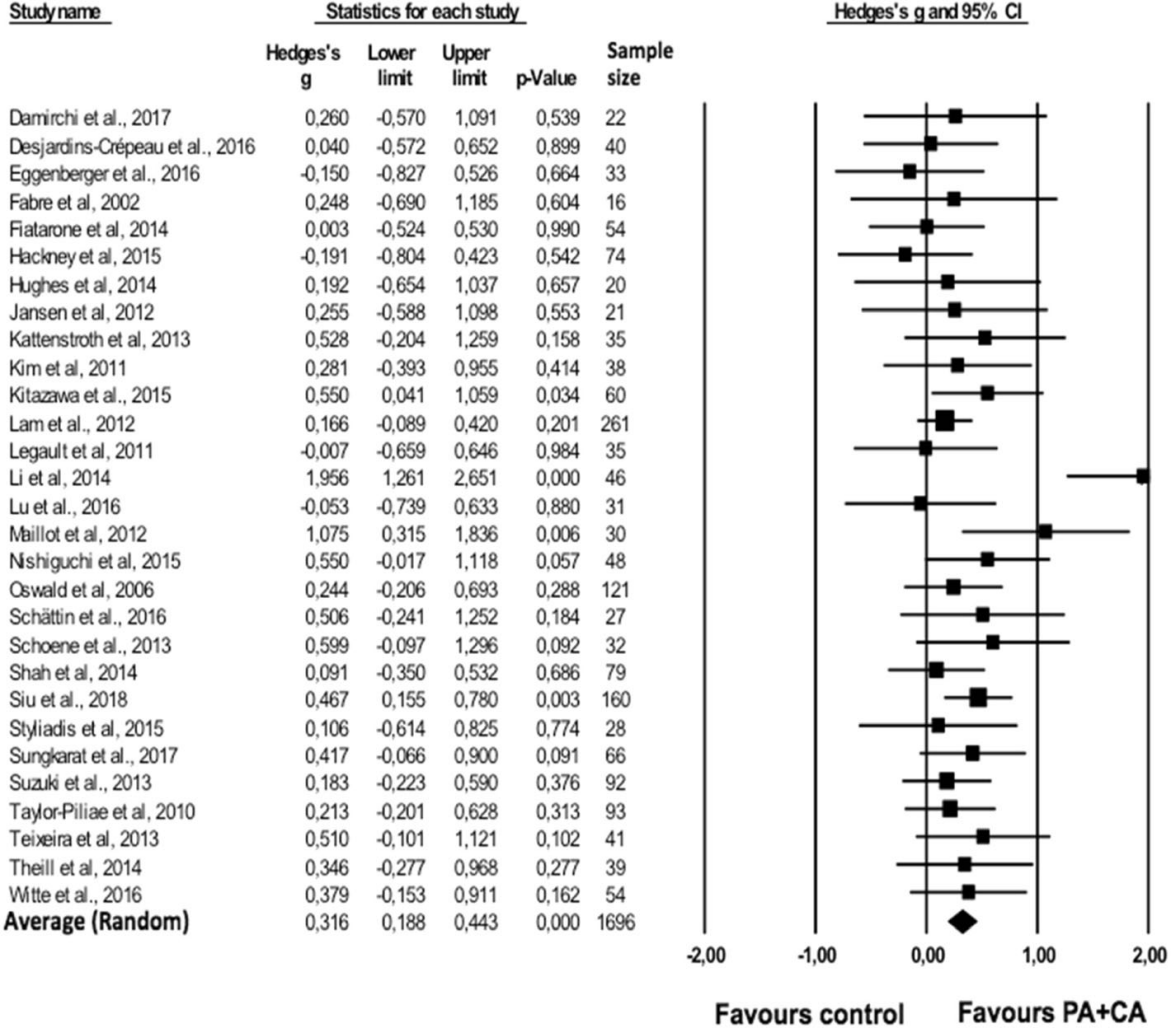
Forest plot of effect sizes for combined PA + CA versus control

For one study [41] effect sizes of all outcomes exceeded the outlier threshold of 3SD above the average effect size. This study was therefore removed from all further analyses. The average effect size across the remaining studies (*n* = 29) indicated that combined PA + CA intervention induced significantly larger gains in cognitive functioning compared to the control intervention (*g* = 0.316; 95% CI 0.188–0.443; *p* < .001) (Fig. 2). Significant heterogeneity was found across studies (*Q*(28) = 41.524; *p* = .048; *I*^*2*^ = 32.569).

#### Combined PA + CA versus PA only group

**Fig. 3:**
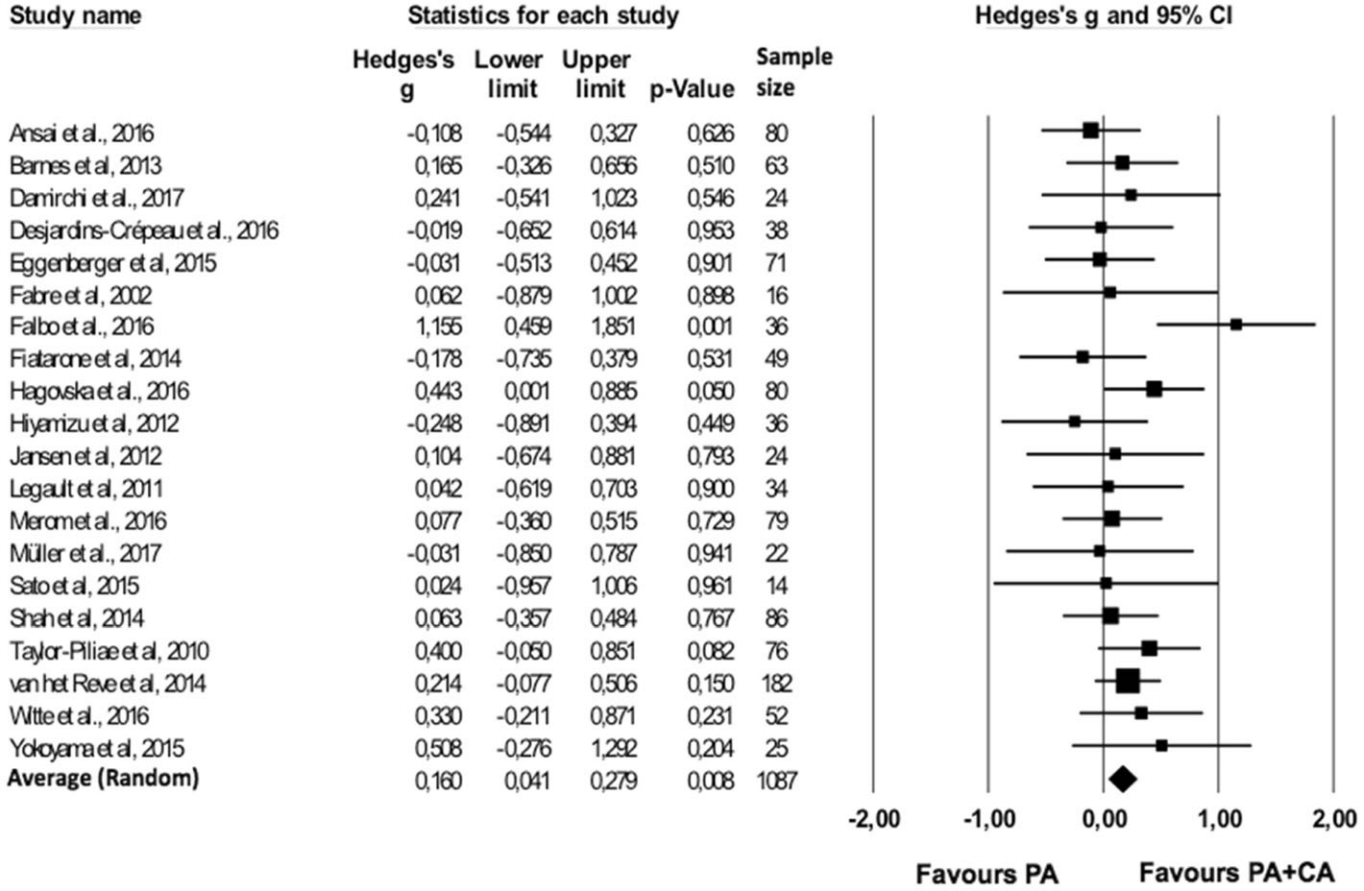
Forest plot of effect sizes for combined PA + CA versus PA only

For one study [41] effect sizes of all outcomes exceeded the outlier threshold (> 3SD above average effect size); this study was therefore removed from all further analyses. For the remaining studies (*n* = 20), the averaged effect size indicated that combined PA + CA interventions induced significantly larger gains in cognitive functioning than the PA interventions alone (*g* = 0.160; 95% CI 0.041–0.279; *p* = .008) (Fig. 3). No significant heterogeneity was found across studies (*Q*(19) = 17.964; *p* = .525; *I*^*2*^ = 0%).

#### Combined PA + CA versus CA only group

**Fig. 4:**
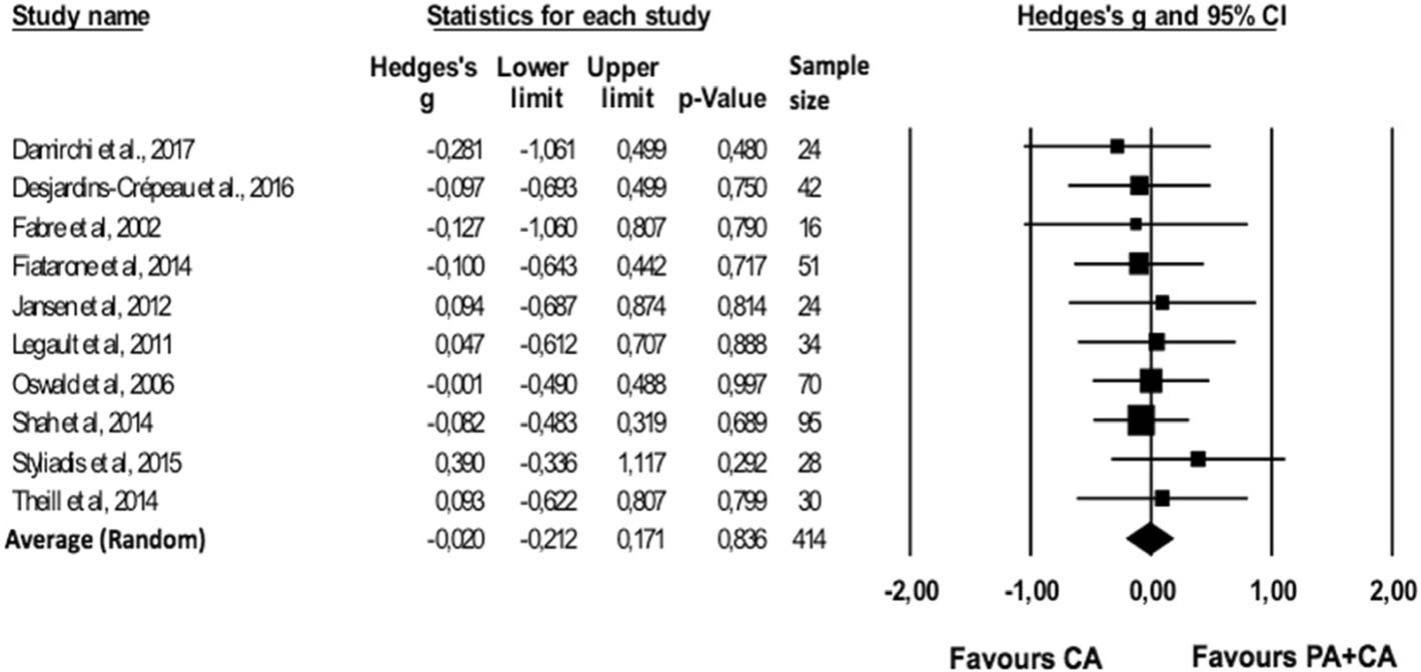
Forest plot of effect sizes for combined PA + CA versus CA only

For two studies [30, 54] the effect size of one outcome exceeded the outlier threshold (> 3SD above average effect size); these outcomes were therefore removed from further analyses. The averaged effect size across the ten studies indicated that overall there were no differences in changes of cognitive functioning between combined PA + CA versus CA interventions alone (*g* = − 0.020; 95% CI -0.212-0.171; *p* = 0.836) (Fig. 4). No significant heterogeneity was found across studies (*Q*(9) = 2.168; *p* = .989; *I*^*2*^ = 0%).

### Sensitivity analyses

The above main analyses were repeated for randomized controlled trials only (i.e., RCTs and cluster-RCTs). Excluding the non-randomized controlled trials did not influence conclusions for all three comparisons (combined PA + CA versus control; combined PA + CA versus PA only; combined PA + CA versus CA only) and revealed similar effect sizes (see Additional file 4).

Since none of the included studies reported exact pre-post test correlation values, the standard approximation of *r* = .50 was used. For all three comparisons, a sensitivity analysis was then conducted for lower (*r* = 0.20) and higher (*r* = 0.80) pre-post test correlation values. This revealed that effect sizes largely remained within the 95% confidence interval. Also, funnel plots and Egger’s Tests indicated that potential publication bias could be ruled out (see Additional file 4).

### Moderator analyses

Because significant heterogeneity was found only for the comparison of combined PA + CA versus control, the effect of potential moderating variables was focused on this comparison only. Table 2 summarizes the results of the moderator analyses. A trend towards significance was found for the mode of PA + CA combination (Q = 3.699; *p* = .054). The largest effects were found for the interventions using a simultaneous design (g = 0.385; 95%CI 0.214–0.555; *p* < .001) and smaller, non-significant effects were found for studies using sequential designs (g = 0.114; 95%CI -0.102- 0.331, *p* = .301). Effects did not significantly depend on the cognitive status of participants (Q = 0.427; *p* = .514). Studies with cognitively healthy and cognitively impaired participants showed significant positive effects (healthy: g = 0.282; 95%CI 0.148–0.416, *p* < .001 and impaired: g = 0.389; 95%CI 0.095–0.684, *p* = .009). Analyses revealed no significant influence of intervention length (Q = 2.235; *p* = .327); session duration (Q = 3.955; *p* = .138), session frequency (Q = 4.398; *p* = .111) or EPHPP study quality scores (Q = 2.080; *p* = .353) (see Additional file 5 for EPHPP quality rating scores).

**Table 2:** Moderator analyses of effect sizes for combined PA + CA versus control

Moderator	n	k	Hedges’g (95%CI)	p	Q	p
Mode of combination^a^	1620	27			3.699	.054
sequential	367	7	0.114 [−0.102; 0.331]	.301		
simultaneous	1253	20	0.385 [0.214; 0.555]	<.001		
Cognitive status	1696	29			0.427	.514
healthy	947	20	0.282 [0.148; 0.416]	<.001		
impaired	749	9	0.389 [0.095; 0.684]	.009		
Intervention length	1696	29			2.235	.327
short (< 12 weeks)	257	8	0.335 [0.092; 0.577]	.007		
medium (12–23 weeks)	725	13	0.405 [0.144; 0.666]	.002		
long (≥ 24 weeks)	714	8	0.194 [0.038; 0.349]	.015		
Session duration^b^	1568	27			3.955	.138
short (≤ 45 min)	433	6	0.248 [0.055; 0.441]	.012		
medium (> 45 to ≤60 min)	679	13	0.474 [0.231; 0.716]	<.001		
long (> 60 min)	456	8	0.159 [−0.041; 0.358]	.12		
Session frequency^c^	1518	25			4.398	.111
low (1×/week)	236	4	0.379 [0.092; 0.667]	.010		
medium (2×/week)	545	9	0.542 [0.217; 0.867]	.001		
high (≥3×/week)	737	12	0.190 [0.043; 0.336]	.011		
EPHPP study quality	1696	29			2.080	.353
weak	560	9	0.280 [0.106; 0.454]	.002		
moderate	537	13	0.454 [0.176; 0.733]	.001		
strong	599	7	0.215 [0.046; 0.383]	.013		

n = combined sample size; k = number of studies; Hedges’g (random effects); *CI* confidence interval, Q = homogeneity statistic (mixed effects)

^a^Two studies were excluded because the mode of PA + CA combination had both sequential and simultaneous components [46, 51]

^b^Two studies were excluded because average session duration (particularly, of home-based sessions) was not clearly reported [21, 40]

^c^Four studies were excluded because session frequency was not clearly reported [32, 36, 40, 46]

## Discussion

This meta-analysis investigated effects of combined physical and cognitive training interventions on the cognitive functioning of older adults. Results from 40 studies were included in final analyses and effect sizes of combined PA + CA intervention were compared to control groups (*n* = 29), groups with PA interventions alone (*n* = 20), and groups with CA interventions alone (*n* = 10). To our knowledge this is the first meta-analysis on this topic using a more comprehensive approach: on top of the typically structured dual-tasking programs we included existing intervention programs having strong intrinsic combination of physical and cognitive training such as dance and tai chi classes. Also, this meta-analysis further explored differences between cognitively healthy older adults and older adults with mild cognitive impairment.

Overall, results indicated that interventions combining physical and cognitive activity could improve cognitive functioning in older adults. Larger effects were found for combined PA + CA versus control and PA interventions alone. Yet, no additive effects were found when comparing combined interventions to CA interventions alone. Gains in cognitive functioning tended to be larger for interventions consisting of simultaneously versus sequentially combined PA + CA. Additionally, the results showed that gains in cognition can be expected following combined PA + CA interventions for both cognitively healthy and mildly impaired older adults.

Our findings support recent views that the human brain retains a lifelong capacity to reorganize and change and that cognitive functioning, even at older age, can be improved [2, 3]. Although the additive effects of combined interventions compared to PA interventions alone were small, they are in line with current theories of enriched environments [67]. Within an enriched environment PA is considered the key trigger for the upregulation of neurotrophic factors and neurogenesis [68, 69]. Furthermore, it has been suggested that additional cognitively demanding conditions are necessary to promote synaptic plasticity and the survival and functional integration of the newly formed neurons into neural networks [70, 71]. In rodents, combining PA (e.g., voluntary wheel-running) with challenging cognitive tasks (e.g., maze training) showed larger and longer-lasting gains in learning and memory abilities relative to single activity interventions [12, 13]. A previous meta-analysis on healthy older adults [16] reported similarly small but significant positive effects for combined PA + CA interventions compared to PA interventions alone. Also, they showed similar null results for the comparison with CA only interventions. A first plausible explanation for this latter finding could be that generally the cognitive effort during simultaneously combined PA + CA is lower compared to CA interventions alone. The effect of cognitive training during simultaneous PA + CA could have been underestimated because neural resources have to be shared during the performance of concurrent cognitive-motor tasks [72]. Hence, synergistic neuroplastic effects following simultaneously combined PA + CA may need more time to establish than following CA interventions alone. Another explanation could be that for the comparison between combined PA + CA versus CA only, the majority of studies in our meta-analysis used a sequential design in which the combined PA + CA groups participated in twice as many sessions than the intervention groups with only CA. In several studies these sessions were even added on the same day [31, 40, 47]. It has been suggested that such high frequencies of training sessions might induce too much stress and fatigue [16]; factors that have been demonstrated to negatively moderate the effects of PA on cognition and neuroplasticity [73]. This potentially negative effect of sequentially designed studies may not have influenced the overall effect size as much in our comparisons between combined PA + CA versus control and PA only, since these latter analyses included relatively less sequentially designed studies. Yet, the above hypotheses for the lack of differential changes in cognitive functioning between combined PA + CA interventions and CA interventions alone remain speculative. Given that this comparison included only 10 studies (of which 6 non-RTCs), there is high need for more well designed studies focusing on this research question. Also, the fact that combined PA + CA interventions in this meta-analysis revealed similar cognitive benefits versus CA interventions alone, does not necessarily call for the promotion of single CA interventions because one should not forget the important physical health benefits following the PA component in the combined interventions.

The idea that observed differences in cognitive effects are (partly) related to the mode of combination has also been claimed before by Zhu et al. (2016) who reported larger effects for simultaneous versus sequential combinations [16]. Yet, the lack of a significant difference in their meta-analysis [16] was suggested to result from limited power. In our current meta-analysis, which included 18 more studies, we did find a trend towards significantly superior benefits for simultaneous versus sequential intervention programs. One potential explanation for superior cognitive effects following simultaneously combined PA + CA interventions is the temporary nature of the increase of peripheral brain-derived neurotrophic factor (BDNF). It has been shown that the BDNF increase returns to baseline 10–60 min after physical activity [23]. This transient nature of BDNF increase calls for a temporarily close succession of CA to optimally benefit from these neurotrophic effects.

Furthermore, moderator analyses showed that effects did not significantly depend on the intervention characteristics such as total intervention length, session duration and session frequency. For all three categories of intervention length (i.e., < 12 weeks, 12–23 weeks, ≥24 weeks) significant positive effects were found. Studies conducting combined PA + CA sessions of a short (≤ 45 min) as well as medium duration (> 45 to ≤60 min) revealed significant effect sizes in comparison with studies with long session durations (> 60 min) that were not statistically significant. Also, although no significant difference in effect size was found for intervention sessions conducted once per week, twice per week, or three or more times per week effect sizes were on average smaller for the highest frequency of delivery. These tentative findings are in line with results from the meta-analysis by Zhu et al. (2016) who reported less efficacy for combined interventions scheduled five or more times per week compared to interventions administrated less than five times per week [16]. Also, a meta-analysis by Northey et al. (2017) [4] and Colcombe & Kramer (2003) [74] on the cognitive effects of physical exercise interventions in older adults concluded that high doses (high frequency, long intervention and long session duration) are not required to produce higher efficacy. Alltogether, current evidence seems to suggest that combined PA + CA interventions of any frequency and duration are beneficial to cognitive functioning. The lack of a dose-response relation contradicts with evidence from epidemiological studies showing higher engagement in cognitive and physical exercise to be associated with better cognitive performance at later age [75]. Convincing statements about dose-response relations however can currently not be made since studies on combined PA + CA explicitly manipulating these factors and investigating broader ranges of session frequency, duration and intervention length are currently lacking.

Our moderation analyses further revealed that differences in cognitive effects were not influenced by the presence/absence of mild cognitive impairment. The fact that cognitive gains can also be expected for older adults who already have some mild cognitive problems is very promising and in line with previous reviews [14, 17, 76]. Mild cognitive impairment is often an intermediate stage in the progression towards dementia and hence, considered a crucial stage with opportunities to intervene in the neurocognitive disease. Combined PA + CA programs could therefore be used as a modality for treating as well as preventing cognitive decline in older adults. In the future, research should explore the feasibility and effects of combined programs in the treatment of more advanced stages of impairment such as dementia.

### Strengths and limitations of the review

The current meta-analysis adds to the existing knowledge on combined PA + CA interventions by updating and extending the literature search, for the first time with the explicit inclusion of more ecologically valid types of combined interventions such as dance and tai chi programs. This is considered a strength for further implementation purposes as it can better inform health actors on which existing activities to promote in order to keep older adults cognitively healthy. However, this also implied considerable heterogeneity among intervention programs with respect to the content and complexity of PA and CA training elements.

A second strength of this meta-analysis is the inclusion of studies on cognitively healthy older adults as well as on populations with mild cognitive impairment. We aimed to explore if any onset of cognitive impairment might attenuate the effect. Therefore, we used broad inclusion criteria for populations with mild cognitive impairment, i.e., assessments for cognitive impairment could be based on both subjective and objective reports. As such, the mildly cognitively impaired subpopulation in our meta-analysis involves a heterogeneous group. A distinction between different subtypes of cognitive impairment was not the purpose of the current study, nor was it feasible given the lack of power.

Another limitation of the included studies is the lack of long-term interventions and follow-ups. Also, methodological details were often missing, so that the influence of other potentially moderating factors could not be determined (e.g., baseline levels of physical/cognitive fitness, physical/cognitive training intensity). Furthermore, cognitive intervention components could include single-domain or multi-domain cognitive training. Moreover, many different cognitive tests were used to measure outcomes on different cognitive functions and could involve trained or untrained tasks (resulting in near or far transfer effects). For instance, some interventions specifically targeted the training of executive functioning [49, 59] or memory [40, 54] whereas other studies trained participants on a mix of executive control, memory, language and visuospatial functions [30, 31, 47, 58]. Some studies evaluated cognitive benefits using measures of global cognition, such as the well known ADAS-cog [39, 52] or MMSE scales [51, 62]. Other studies evaluated changes specifically on executive control using e.g. the Trail making test [27, 44, 61] or visual learning using e.g. the Rey Auditory Verbal learning test [26, 27, 50] or memory function using different versions of digit/visual/spatial span tests [30, 44, 54]. Especially for the different dance and tai chi interventions, clear information on the trained cognitive domains was lacking and therefore did not allow critical analyses of the effects on different cognitive subfunctions.

## Conclusions

Evidence from this meta-analysis suggests that PA programs for older adults can yield superior cognitive benefits when cognitive tasks are integrated into the programs. Consequently, older adults should be made aware of the plastic properties of their brain, the potential to maintain/improve their cognitive functioning and the importance to engage in mentally challenging physical activity (e.g. learn new routes for neighborhood walks). Also, the promotion of activities that intrinsically combine PA and CA (e.g. dance, tai-chi) should receive more attention. Overall, more research resources should be invested in further unravelling dose-response and lasting effects; to identify the optimal programs that maximally take advantage of the neuroplastic properties of the human brain.

## Additional files

Additional file 1 : The PRISMA checklist (DOCX 37 kb)

Additional file 2 : The study protocol (DOCX 37 kb)

Additional file 3 : EPHPP quality assessment tool (DOCX 91 kb)

Additional file 4 : Sensitivity analyses (DOCX 30 kb)

Additional file 5 : EPHPP quality rating scores (DOCX 38 kb)

## Abbreviations

CA: Cognitive activity
CMA: Comprehensive meta-analysis software
EPHPP: Effective public health practice project
MCI: Mild cognitive impairment
MMSE: Mini-mental state examination
PA: Physical activity
PRISMA: Preferred reporting items for systematic reviews and meta-analysis
RCT: Randomized controlled trial
SD: Standard deviation
