# Supplementary material for: Physical activity to improve cognition in older adults: can physical activity programs enriched with cognitive challenges enhance the effects? A systematic review and meta-analysis
Source: Int J Behav Nutr Phys Act. 2018 Jul 4;15:63. doi: 10.1186/s12966-018-0697-x (PMC6032764; doi:10.1186/s12966-018-0697-x)
Supplement: Supplementary file 2 — : The study protocol (DOCX 37 kb) [file 12966_2018_697_MOESM2_ESM.docx]

**Additional file 2: The study protocol**

**Objectives**

To investigate the efficacy of combined physical activity (PA) and cognitive activity (CA) interventions on cognitive functioning in older adults. To investigate differences in cognitive gains following combined PA+CA versus interventions including PA only, CA only or control.

To identify factors moderating the observed efficacy:

1. Mode of combination: sequentially versus simultaneously combined PA+CA
2. Cognitive status of the participants: cognitively healthy or mildly cognitively impaired
3. Intervention length
4. Session duration
5. Session frequency
6. Study quality

**Eligibility criteria for including studies**

1. Type of studies

Only controlled intervention trials were included (RCT: randomly assigning individuals to intervention and comparison groups), cluster-RCTs (randomly assigning groups to intervention and comparison groups) and non-randomised controlled trials (non-RCT; assigning subjects using methods that are not random, e.g. (partly) based on preference). These studies included pre-post measurements for which an effect size could be calculated on objective cognitive outcome measures. Studies evaluating the effects of single bouts of exercise were not considered.

1. Type of participants

Independently living older adults (overall mean age ≥ 65 years) with or without mild cognitive impairment at baseline but without dementia, and without other mental or neurological disease (e.g. stroke, depression, parkinson). Assessment of mild cognitive impairment could be based on subjective and/or objective tests. If the overall mean age was not reported and mean age of groups was reported, the overall mean was calculated ((M1*N1) + (M2*N2)) /(N1+N2).

1. Type of interventions

*Combined PA+CA intervention:* the combination of a physical activity and cognitive activity intervention could be

- *sequential*: separate sessions of PA before or after separate sessions of CA.

PA sessions must involve aerobic training (e.g., walking, cycling) or strength training or a combination of both. PA sessions can also include additional balance or stretching exercises but studies were only eligible as long as the PA intervention component included at least an aerobic or strength training component. CA sessions must involve cognitive training exercises aimed to train single or multiple domains of cognitive function.

or

- *simultaneous*: sessions including PA and CA simultaneously through e.g. exergames, dual-task exercises, dance, tai chi or related martial arts. The same eligibility criteria for the PA and CA hold as for the sequential PA and CA sessions described above: the PA component includes at least aerobic or strength exercises and the CA component can involve training of single or multiple cognitive functions. Simultaneous PA+CA interventions were categorized into the following programs:

- *Exergames*: exercise based computer games: virtual reality-enhanced exercise combining PA with computer-simulated, interactive environments comprising cognitive stimulation

- *Dual-tasks:* tasks that require simultaneous performance of physical and cognitive exercises, e.g. learning a list of new words while walking.

- *Dance:* learning new dance steps requiring both PA components (e.g. aerobic performance, motor coordination) and CA components (e.g. memory, attention).

- *Tai chi or related martial arts:* classes incorporating aerobic and mobility exercises and training in sustained attention, multitasking and learning of complex movement patterns stimulating visuo-spatial processing and episodic memory.

A multitude of styles are available for tai chi and dance, each style emphasizing particular components. Similarly, many different types of exergames are available for older adults. Although these activities are recognized as moderate aerobic physical activities (Ainsworth et al., 2011), not every style or game necessarily comprises a clear emphasis on specific cognitive training components. Therefore, given the purpose of the current meta-analysis, only studies were included when the tai chi, dance intervention or exergame was intentionally set up as a multimodal physically and cognitively *effortful* intervention (i.e., the intervention is introduced/discussed as a combined physical and cognitive activity and/or the cognitive components are described in the method section).

Studies were excluded when the combined PA+CA intervention included an additional lifestyle intervention (e.g. diet or dietary supplements, psychological group counseling, educational sessions on how to change nutrition habits).

*Comparison interventions:* Studies were only considered when at least one of the following comparison groups was included: active or passive *control group* (e.g. no intervention/usual care or including health education/social/stretching classes i.e., not comprising explicit physical training aimed to improve aerobic performance or strength and not comprising explicit cognitive training intended to improve cognitive function) or *PA only group* (intervention including only physical exercises, no cognitive training) or *CA only group* (intervention including only cognitive training, no physical exercises).

If one of the comparison groups was potentially biased (e.g. the PA only group also engages in some cognitive training or the CA only group also engages in physical (aerobic or strength) training, comparison with this particular group was not included in the meta-analysis.

When both an active and passive control group where tested, preference was given to data input from the active control group to allow better control of the amount of social stimulation.

1. Type of outcome measure

Cognitive outcome measures included pre and post intervention scores on objective cognitive tests. Subjective measures (e.g., questionnaires on activities of daily living) and non single cognitive outcomes (e.g., motor-cognitive dual-task outcomes from which effects on sole cognitive functions cannot be disentangled) were excluded from the analysis. When results from cognitive tests were presented both for composite battery scores as well as for the separate tests from the battery (e.g. for MMSE, ADAS-cog, MOCA), only the separate outcomes were included in CMA.

Studies for which an effect size could be calculated only for the reported significant effects were excluded to avoid bias, when no information could be obtained upon request from authors on non-significant results. When assessments were conducted at interim time points, only post-test at latest end-point of intervention was included. Follow-up assessments, after the intervention had ended, were not considered.

**Search strategy**

1. Information sources

Published studies were identified using the electronic databases Pubmed, Embase, PsycInfo, CINAHL and Sportdiscus. References and citation lists of papers and published reviews were searched. The search was limited to papers written in English.

1. Search terms

Because there is no consistent use of terminology to define combined PA+CA interventions, a broad range of search terms was used:

(((physical OR aerobic OR endurance OR cardiorespiratory OR cardiovascular OR resistance OR strength)

AND

(cognitive OR mental OR mind))

OR

(multimodal OR multidomain OR multicomponent OR “multi-modal” OR “multi-domain” OR “multi-component” OR “dual task” OR “dual-task” OR “tai chi” OR “taiji” OR “tai chi chuan” OR danc* OR exergam*))

AND

(training OR activity OR intervention OR stimulation OR program OR exercise)

AND

(aging OR ageing OR elderly OR elders OR seniors OR older adults OR “mild cognitive impairment”)

AND

(cognition OR cognitive OR memory OR “executive function*” OR “executive control” OR attention OR visuospatial OR “processing speed” OR language)

**Methods of the review**

1. Selection of the studies

Initial screening based on title and abstract was performed by the first author (FG). Full texts after this first selection were screened independently by two reviewers (FG and LP) in accordance with the inclusion and exclusion criteria set in the study protocol. Consensus was used to resolve disagreement regarding inclusion of the studies. When doubt regarding study eligibility persisted, this was resolved by a third reviewer (WF).

1. Data extraction

Data extraction was done by FG and LP independently. Consensus was used to resolve disagreement regarding the coding categories. When doubt regarding coding persisted, this was resolved by a third reviewer (WF).

Cognitive outcome measures

For the meta-analyses, cognitive outcome data were extracted in the form of means and standard deviations of each group for both pre and post assessment (or mean changes and SD differences). For each study an effect size was calculated with Hedges’ formula correcting for small samples (Hedges, 1981). For studies which used multiple dependent intervention groups within one study (e.g. several similar intervention groups and one control condition), the multiple groups were combined using the following formula’s (Higgings and Green, 2011):

|  | Group 1 | Group 2 | Combined groups |
| --- | --- | --- | --- |
| Sample size | N_1_ | N_2_ | N_1_ + N_2_ |
| Mean | M_1_ | M_2_ | (N_1_M_1_ + N_2_M_2_) / (N_1_+N_2_) |
| SD | SD_1_ | SD_2_ | √ (((N_1_-1)SD_1_² + (N_2_-1)SD_2_² +((N_1_N_2_)/(N_1_+N_2_)) (M_1_²+M_2_²-2M_1_M_2_)) / N_1_+N_2_ -1) |

Quality assessment

By using the Quality Assessment tool for Quantitative Studies from the Effective Public Health Practice Project (EPHPP, http://www.ephpp.ca/PDF/Quality%20Assessment%20Tool_2010_2.pdf), the methodological quality of the separate studies was evaluated. This was done independently by 2 reviewers (LP and AD). When there was a discrepancy between the two independent reviewers’ score, a third reviewer (FG) was involved to resolve the discrepancy and to reach a consensus. Analyses were performed to assess whether the quality of study impacted the outcomes.

Moderator variables

Potential moderator variables were extracted from each study independently by 2 reviewers (FG and LP). The coding for session duration (i.e. average minutes duration of a single session, including any warm-up and cool-down), session frequency (i.e. number of sessions per week) and intervention length (i.e. total number of intervention weeks) for the combined PA+CA intervention was based on previous reviews (Colcombe and Kramer, 2003; Northey et al., 2017) and adapted for better fit on the dataset. Session duration was categorized as short (≤ 45min), medium (>45 to ≤60 min) and long (> 60 min). Session frequency was coded as low (1 session/week), medium (2 sessions/week) or high (≥ 3 sessions/week). Intervention length was coded as short (<12 weeks), medium (12 – 23 weeks), or long (≥ 24 weeks).

1. Data analyses

Comprehensive Meta-analysis (CMA) software version 3.3.070 (Biostat Inc., Englewood, NJ, USA) was used to compute the effect sizes and conduct all analyses. For each study, effect sizes were averaged across all cognitive measures to determine the effect of combined PA+CA intervention versus the comparison groups on overall cognition. Random effects models were used with a positive Hedges' g or a negative Hedges' g indicating that the combined PA+CA intervention induced respectively higher or lower gains in cognition versus the comparison intervention. In cases where the cognitive outcome measure was negatively scored (higher scores reflect decline of cognitive function), the computed sign of the effect size was reversed so all positive differences reflected a higher improvement in cognition for the combined PA+CA intervention. Heterogeneity between studies was assessed using the Cochran’s Q-value and I^2^ statistic with a significant p value indicating large variability of effect sizes between studies. Moderator analyses were conducted to test whether the heterogeneity can be explained by differences in methodological variables (moderator variables, described above).

1. Sensitivity analyses

Sensitivity analyses were performed to identify possible outliers (mean Hedges’ g effect size ± 3SD). Also, main analyses were repeated to make sure the results and conclusions were not dependent on specific methodological decisions and assumptions. All analyses (combined PA+CA versus control; combined PA+CA versus PA only; combined PA+CA versus CA only) were repeated for randomized controlled trials only (i.e., excluding non-randomized controlled trials) and for pre–posttest correlations set at lower (0.20) and higher (0.80) values than the standard assumption of 0.50. Finally, potential publication bias was evaluated via a funnel plot and Egger’s regression test.

References:

Ainsworth BE, Haskell WL, Herrmann SD, Meckes N, Bassett Jr DR, Tudor-Locke C, Greer JL, Vezina J, Whitt- Glover MC, Leon AS. 2011 Compendium of Physical Activities: a second update of codes and MET values. Medicine and Science in Sports and Exercise, 2011;43(8):1575-1581.

Colcombe S, Kramer AF. Fitness effects on the cognitive function of older adults: a meta-analytic study. Psychol Sci. 2003;14(2):125–30.

Hedges L: Distribution Theory for Glass's Estimator of Effect Size and Related Estimators. *Journal of Educational Statistics* 1981, 6: 128.

Higgings JPT, Green S: *Cochrane Handbook for Systematic Reviews of Interventions*, Version 5.1.0 edn. The Cochrane Collaboration; 2011.

Northey JM, Cherbuin N, Pumpa KL, Smee DJ, Rattray B. Exercise interventions for cognitive function in adults older than 50: a systematic review with meta-analysis. Br J Sports Med. 2017 Apr; 0: 1-9.
