# Supplementary material for: Physical activity to improve cognition in older adults: can physical activity programs enriched with cognitive challenges enhance the effects? A systematic review and meta-analysis
Source: Int J Behav Nutr Phys Act. 2018 Jul 4;15:63. doi: 10.1186/s12966-018-0697-x (PMC6032764; doi:10.1186/s12966-018-0697-x)
Supplement: Supplementary file 3 — : EPHPP quality assessment tool (DOCX 91 kb) [file 12966_2018_697_MOESM3_ESM.docx]

**Additional file 3: EPHPP quality assessment tool (**[**https://merst.ca/ephpp/**](https://merst.ca/ephpp/)**)**

**COMPONENT RATINGS**

**A) SELECTION BIAS**

**(Q1) Are the individuals selected to participate in the study likely to be representative of the target population?**

1. Very likely
2. Somewhat likely
3. Not likely
4. Can’t tell

**(Q2) What percentage of selected individuals agreed to participate?**

1. 80 - 100% agreement
2. 60 – 79% agreement
3. less than 60% agreement
4. Not applicable
5. Can’t tell

| **RATE THIS SECTION** | **STRONG** | **MODERATE** | **WEAK** |
| --- | --- | --- | --- |
| See dictionary | 1 | 2 | 3 |

**B) STUDY DESIGN**

**Indicate the study design**

1. Randomized controlled trial
2. Controlled clinical trial
3. Cohort analytic (two group pre + post)
4. Case-control
5. Cohort (one group pre + post (before and after))
6. Interrupted time series
7. Other specify ____________________________
8. Can’t tell

**Was the study described as randomized? If NO, go to Component C.**

No Yes

**If Yes, was the method of randomization described? (See dictionary)**

No Yes

**If Yes, was the method appropriate? (See dictionary)**

| No Yes **RATE THIS SECTION** | **STRONG** | **MODERATE** | **WEAK** |
| --- | --- | --- | --- |
| **See dictionary** | 1 | 2 | 3 |

**C) CONFOUNDERS**

**(Q1) Were there important differences between groups prior to the intervention?**

1. Yes
2. No
3. Can’t tell

**The following are examples of confounders:**

1. Race
2. Sex
3. Marital status/family
4. Age
5. SES (income or class)
6. Education
7. Health status
8. Pre-intervention score on outcome measure

**(Q2) If yes, indicate the percentage of relevant confounders that were controlled (either in the design (e.g. stratification, matching) or analysis)?**

1. 80 – 100% (most)
2. 60 – 79% (some)
3. Less than 60% (few or none)
4. Can’t Tell

| **RATE THIS SECTION** | **STRONG** | **MODERATE** | **WEAK** |
| --- | --- | --- | --- |
| **See dictionary** | 1 | 2 | 3 |

**D) BLINDING**

**(Q1) Was (were) the outcome assessor(s) aware of the intervention or exposure status of participants?**

1. Yes
2. No
3. Can’t tell

**(Q2) Were the study participants aware of the research question?**

1. Yes
2. No
3. Can’t tell

| **RATE THIS SECTION** | **STRONG** | **MODERATE** | **WEAK** |
| --- | --- | --- | --- |
| **See dictionary** | 1 | 2 | 3 |

**E) DATA COLLECTION METHODS**

**(Q1) Were data collection tools shown to be valid?**

1. Yes
2. No
3. Can’t tell

**(Q2) Were data collection tools shown to be reliable?**

1. Yes
2. No
3. Can’t tell

| **RATE THIS SECTION** | **STRONG** | **MODERATE** | **WEAK** |
| --- | --- | --- | --- |
| **See dictionary** | 1 | 2 | 3 |

**F) WITHDRAWALS AND DROP-OUTS**

**(Q1) Were withdrawals and drop-outs reported in terms of numbers and/or reasons per group?**

1. Yes
2. No
3. Can’t tell
4. Not Applicable (i.e. one time surveys or interviews)

**(Q2) Indicate the percentage of participants completing the study. (If the percentage differs by groups, record the lowest).**

1. 80-100%
2. 60-79%
3. Less than 60%
4. Can’t tell
5. Not applicable (i.e. Retrospective case-control)

| **RATE THIS SECTION** | **STRONG** | **MODERATE** | **WEAK** |
| --- | --- | --- | --- |
| **See dictionary** | 1 | 2 | 3 |

**GLOBAL RATING**

**COMPONENT RATINGS**

Please transcribe the information from the gray boxes on pages 1-4 onto this page. See dictionary on how to rate this section.

**
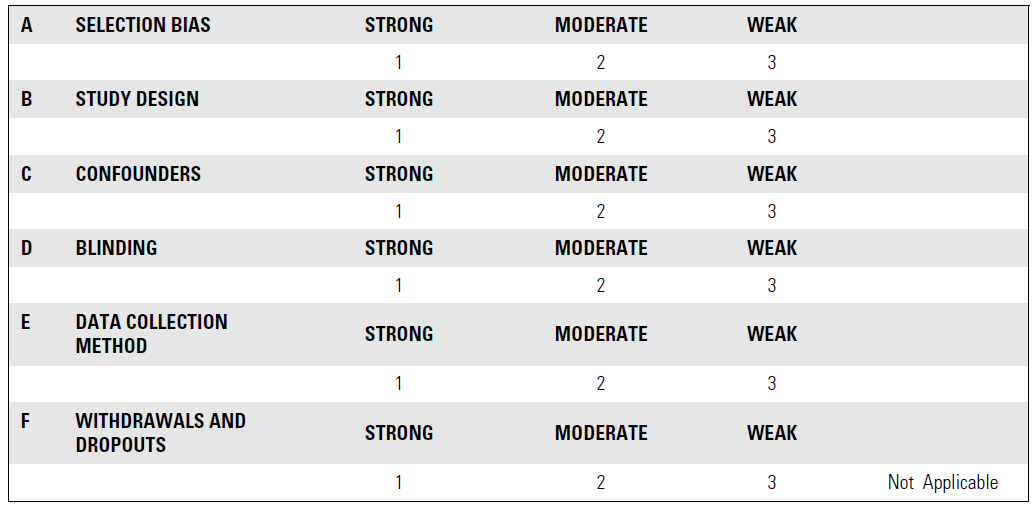
**

**GLOBAL RATING FOR THIS PAPER (circle one):**

1 STRONG (no WEAK ratings)

2 MODERATE (one WEAK rating)

3 WEAK (two or more WEAK ratings
