# Supplementary material for: Physical activity to improve cognition in older adults: can physical activity programs enriched with cognitive challenges enhance the effects? A systematic review and meta-analysis
Source: Int J Behav Nutr Phys Act. 2018 Jul 4;15:63. doi: 10.1186/s12966-018-0697-x (PMC6032764; doi:10.1186/s12966-018-0697-x)
Supplement: Supplementary file 4 — : Sensitivity analyses (DOCX 30 kb) [file 12966_2018_697_MOESM4_ESM.docx]

**Additional file 4: Sensitivity analyses: effects of excluding non-randomized controlled trials, effects of pre-post test correlation values and Egger’s regression test for publication bias**

|  | Hedges’ g (95% CI) | p | t |
| --- | --- | --- | --- |
| Combined PA+CA vs Control |  |  |  |
| Average effect size | 0.316 [0.188; 0.443] | *<.001* |  |
| Average effect size, non-RCTs excluded | 0.290 [0.176; 0.404] | *<.001* |  |
| Pre-post test correlation |  |  |  |
| - At r=.20 | 0.255 [0.151; 0.360] | *<.001* |  |
| - At r=.80 | 0.457 [0.283; 0.631] | *<.001* |  |
| Egger’s Test |  | *.394* | 0.866 |
| Combined PA+CA vs PA |  |  |  |
| Average effect size | 0.160 [0.041; 0.279] | *.008* |  |
| Average effect size, non-RCTs excluded | 0.169 [0.040; 0.299] | *.010* |  |
| Pre-post test correlation |  |  |  |
| - At r=.20 | 0.143 [0.024; 0.261] | *.018* |  |
| - At r=.80 | 0.195 [0.049; 0.340] | *.009* |  |
| Egger’s Test |  | *.983* | 0.022 |
| Combined PA+CA vs CA |  |  |  |
| Average effect size | -0.020 [-0.212; 0.171] | *.836* |  |
| Average effect size, non-RCTs excluded | -0.098 [-0.396; 0.199] | *.517* |  |
| Pre-post test correlation |  |  |  |
| - At r=.20 | -0.017 [-0.209; 0.174] | *.859* |  |
| - At r=.80 | -0.028 [-0.220; 0.165] | *.780* |  |
| Egger’s Test |  | *.527* | 0.661 |

Hedges’ g (random effects); CI= confidence interval; Egger’s regression test (p value two-tailed); non-RCT=non-randomized controlled trials
