# Supplementary material for: Physical activity to improve cognition in older adults: can physical activity programs enriched with cognitive challenges enhance the effects? A systematic review and meta-analysis
Source: Int J Behav Nutr Phys Act. 2018 Jul 4;15:63. doi: 10.1186/s12966-018-0697-x (PMC6032764; doi:10.1186/s12966-018-0697-x)
Supplement: Supplementary file 5 — : EPHPP quality rating scores (DOCX 38 kb) [file 12966_2018_697_MOESM5_ESM.docx]

**Additional file 5: EPHPP quality rating scores**

| Study | Selection  bias | Study design | Confounders | Blinding | Data collection method | Withdrawals and dropouts | Global quality  rating |
| --- | --- | --- | --- | --- | --- | --- | --- |
| Fabre et al. 2002 | weak | strong | strong | moderate | strong | weak | weak |
| Oswald et al. 2006 | weak | moderate | weak | moderate | moderate | weak | weak |
| Taylor-Piliae et al. 2010 | weak | strong | weak | moderate | strong | strong | weak |
| Kim et al. 2011 | moderate | strong | strong | moderate | strong | strong | strong |
| Legault et al. 2011 | weak | strong | strong | weak | strong | strong | weak |
| Hiyamizu et al. 2012 | moderate | strong | strong | moderate | strong | strong | strong |
| Jansen et al. 2012 | weak | moderate | strong | moderate | moderate | strong | moderate |
| Maillot et al. 2012 | weak | strong | strong | moderate | strong | strong | moderate |
| Lam et al. 2012 | moderate | strong | strong | moderate | strong | moderate | strong |
| Barnes et al. 2013 | weak | strong | strong | strong | strong | moderate | moderate |
| Kattenstroth et al. 2013 | weak | strong | weak | moderate | strong | weak | weak |
| Schoene et al. 2013 | weak | strong | strong | moderate | strong | strong | moderate |
| Suzuki et al. 2013 | moderate | strong | strong | moderate | strong | strong | strong |
| Theill et al. 2013 | weak | weak | weak | moderate | strong | strong | weak |
| Teixeira et al. 2013 | weak | strong | strong | moderate | strong | strong | moderate |
| Fiatarone et al. 2014 | weak | strong | strong | strong | strong | strong | moderate |
| Hughes et al. 2014 | strong | strong | strong | moderate | strong | strong | strong |
| Shah et al. 2014 | weak | moderate | strong | moderate | strong | moderate | moderate |
| van het Reve et al. 2014 | moderate | strong | weak | weak | strong | strong | weak |
| Li et al. 2014 | weak | strong | strong | moderate | strong | strong | moderate |
| Hackney et al. 2015 | moderate | strong | strong | moderate | moderate | moderate | strong |
| Eggenberger et al. 2015 | weak | strong | weak | moderate | strong | moderate | weak |
| Yokoyama et al. 2015 | weak | strong | strong | moderate | strong | strong | moderate |
| Sato et al. 2015 | weak | strong | strong | moderate | strong | moderate | moderate |
| Nishiguchi et al. 2015 | moderate | strong | strong | moderate | strong | strong | strong |
| Styliadis et al. 2015 | weak | strong | weak | moderate | strong | strong | weak |
| Kitazawa et al. 2015 | weak | strong | strong | moderate | strong | strong | moderate |
| Leon et al. 2015 | weak | strong | strong | moderate | moderate | strong | moderate |
| Ansai et al. 2016 | weak | strong | strong | weak | strong | weak | weak |
| Desjardins-Crépeau et al. 2016 | weak | strong | strong | moderate | strong | moderate | moderate |
| Eggenberger et al. 2016 | weak | strong | weak | moderate | strong | moderate | weak |
| Falbo et al. 2016 | moderate | strong | strong | moderate | strong | moderate | strong |
| Hagovska et al. 2016 | moderate | strong | strong | strong | strong | strong | strong |
| Lu et al. 2016 | weak | strong | strong | moderate | strong | strong | moderate |
| Witte et al. 2016 | weak | strong | strong | moderate | moderate | strong | moderate |
| Merom et al. 2016 | weak | strong | strong | moderate | strong | moderate | moderate |
| Schättin et al. 2016 | weak | strong | strong | moderate | strong | strong | moderate |
| Müller et al. 2017 | moderate | strong | strong | weak | strong | weak | weak |
| Sungkarat et al. 2017 | moderate | strong | strong | moderate | strong | strong | strong |
| Damirchi et al. 2018 | moderate | strong | strong | weak | strong | strong | moderate |
| Siu et al. 2018 | moderate | strong | weak | weak | strong | strong | weak |

|  |
| --- |
|  |
|  |
|  |
|  |
